# Muscarinic Receptors and BK Channels Are Affected by Lipid Raft Disruption of Salivary Gland Cells

**DOI:** 10.3390/ijms22094780

**Published:** 2021-04-30

**Authors:** Jisoo Lee, Yoon-Jung Kim, La-Mee Choi, Keimin Lee, Hee-Kyung Park, Se-Young Choi

**Affiliations:** 1 Department of Physiology, Dental Research Institute, Seoul National University School of Dentistry, Seoul 03080, Korea; jisulee@snu.ac.kr (J.L.); agi@snu.ac.kr (Y.-J.K.); friend0508@hanmail.net (L.-M.C.); keimin@naver.com (K.L.); 2 DSC Investment, Seoul 04779, Korea; 3 Department of Oral Medicine and Oral Diagnosis, Dental Research Institute, Seoul National University School of Dentistry, Seoul 03080, Korea

**Keywords:** lipid raft, salivary gland, G-protein coupled receptor, BK channel, aquaporin-5, methyl-beta-cyclodextrin

## Abstract

Activity-dependent fluid secretion is the most important physiological function of salivary glands and is regulated via muscarinic receptor signaling. Lipid rafts are important for G-protein coupled receptor (GPCR) signaling and ion channels in plasma membranes. However, it is not well understood whether lipid raft disruption affects all membrane events or only specific functions in muscarinic receptor-mediated water secretion in salivary gland cells. We investigated the effects of lipid raft disruption on the major membrane events of muscarinic transcellular water movement in human salivary gland (HSG) cells. We found that incubation with methyl-β-cyclodextrin (MβCD), which depletes lipid rafts, inhibited muscarinic receptor-mediated Ca^2+^ signaling in HSG cells and isolated mouse submandibular acinar cells. However, MβCD did not inhibit a Ca^2+^ increase induced by thapsigargin, which activates store-operated Ca^2+^ entry (SOCE). Interestingly, MβCD increased the activity of the large-conductance Ca^2+^-activated K^+^ channel (BK channel). Finally, we found that MβCD did not directly affect the translocation of aquaporin-5 (AQP5) into the plasma membrane. Our results suggest that lipid rafts maintain muscarinic Ca^2+^ signaling at the receptor level without directly affecting the activation of SOCE induced by intracellular Ca^2+^ pool depletion or the translocation of AQP5 into the plasma membrane.

## 1. Introduction

Activity-dependent water secretion in exocrine glands is controlled precisely by external signals [1,2]. In particular, the regulation of aquaporin function, a type of water channel, has been studied intensively as a key mechanism of water secretion [3,4,5,6]. Exocrine gland cells use G-protein coupled receptors (GPCRs) to accept extracellular signals and regulate the translocation of aquaporin into the plasma membrane. For example, vasopressin receptors in renal-collecting duct cells induce cyclic adenosine monophosphate (cAMP) production, leading to the membrane translocation of aquaporin-2 (AQP2)/aquaporin-3 (AQP3) [5,7]. In the salivary gland, the Ca^2+^ signaling of GPCRs, including the muscarinic M3 receptor, induces membrane translocation of aquaporin-5 (AQP5) [8,9].

The GPCR activation and subsequent translocation of aquaporin require well-organized signaling pathways that function based on environmental cues. The lipid raft is a cell membrane microdomain composed of cholesterol, glycolipids, sphingolipids, and specific proteins, which acts as the main center of signal transmission [10,11]. Lipid rafts play an important role in various cellular processes, such as signal transduction, protein trafficking, exocytosis, endocytosis, chemotaxis, and cell migration [12]. Particularly in cells with apical-basolateral polarity, lipid rafts have a significant impact on physiological functions [13]. These lipid rafts also contain receptors and ion channels [11,14]. Therefore, lipid rafts regulate various functions mediated by GPCRs, such as vasopressin receptors and muscarinic receptors.

In salivary gland cells, muscarinic receptor-mediated water movement is largely mediated by three membrane events. The first is the activation of the muscarinic receptor GPCR. The second is the store-operated Ca^2+^ entry (SOCE) after cytosolic Ca^2+^ pool depletion [15,16] and the subsequent activation of Ca^2+^-activated ion channels controlling the membrane potential [17]. The third is the cytosolic Ca^2+^-dependent translocation of AQP5 into the plasma membrane [18,19]. Lipid rafts are considered to modulate these steps significantly; however, the role of lipid rafts in each step is not yet clearly understood, even though the role of lipid rafts in vasopressin receptor-mediated AQP2 translocation in the kidney has been relatively well studied. 

In this report, we sought to determine the lipid raft dependency of each activity-dependent AQP5 translocation step in salivary gland cells. Methyl-β-cyclodextrin (MβCD) disrupts lipid rafts by scavenging cholesterol [14,20]. We investigated the effects of MβCD on muscarinic M3 receptor signaling and its downstream membrane events in human salivary gland cells.

## 2. Results

### 2.1. MβCD Preincubation Induced Cholesterol Depletion in HSG Cells

**Figure 1:**
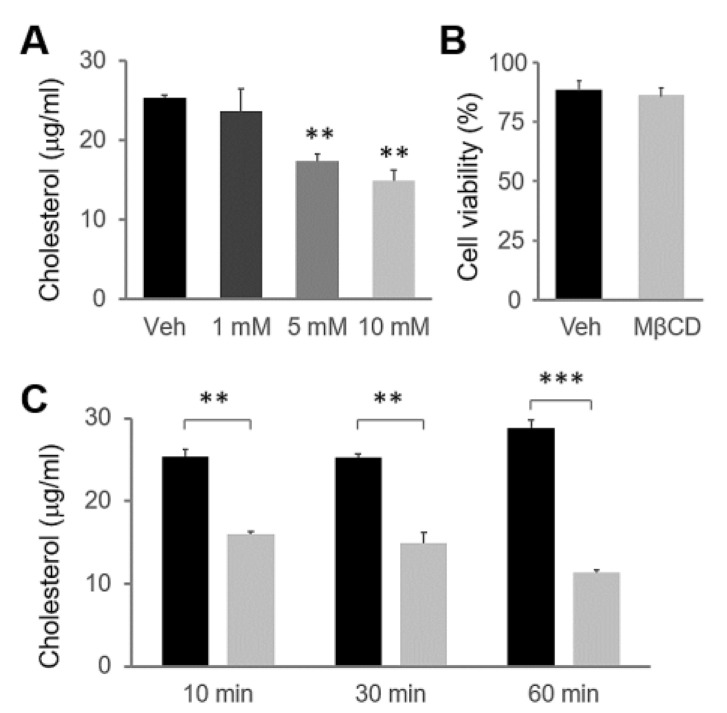
MβCD depletes cholesterol in HSG cells. (**A**) HSG cells were incubated with the indicated concentration of MβCD for 30 min. (**B**) HSG cells were incubated with 10 mM MβCD for 30 min and then tested for cell viability using trypan blue assay. (**C**) The HSG cells were incubated with 10 mM MβCD for the indicated preincubation time. The cholesterol content of cell lysates is depicted as a percentage of the vehicle-treated control (*n* = 3). ** *p* < 0.01; *** *p* < 0.001.

Ca^2+^ signaling and aquaporin translocation in the HSG cell line have been used to study endocrine secretion mechanisms [19,21,22]. Therefore, we first confirmed whether MβCD treatment depletes cholesterol in HSG cells. The previous studies of the concentration-dependent manner of MβCD have revealed that 10 mM MβCD is enough for cholesterol depletion in M07e cells (human leukemia megakaryocytic cell line) [23] and B103 neuroblastoma cells [24]. Comparing cholesterol content at different MβCD concentrations showed that treatment with 10 mM MβCD for 30 min decreased the amount of cholesterol in HSG cells (F_(3,8)_ = 9.51, *p* < 0.01; Figure 1A). The incubation of HSG cells with 10 mM MβCD for 30 min did not affect cell viability (t_(4)_ = 0.36, *p* = 0.73; Figure 1B). We tested the incubation time course of MβCD and found that the most effective treatment time was 30–60 min (F_(5,12)_ = 75.83, *p* < 0.001; Figure 1C).

### 2.2. MβCD Preincubation-Inhibited Muscarinic [Ca^2+^]_i_ Increases without Any Effect on Thapsigargin-Mediated [Ca^2+^]_i_ in Salivary Gland Cells

**Figure 2:**
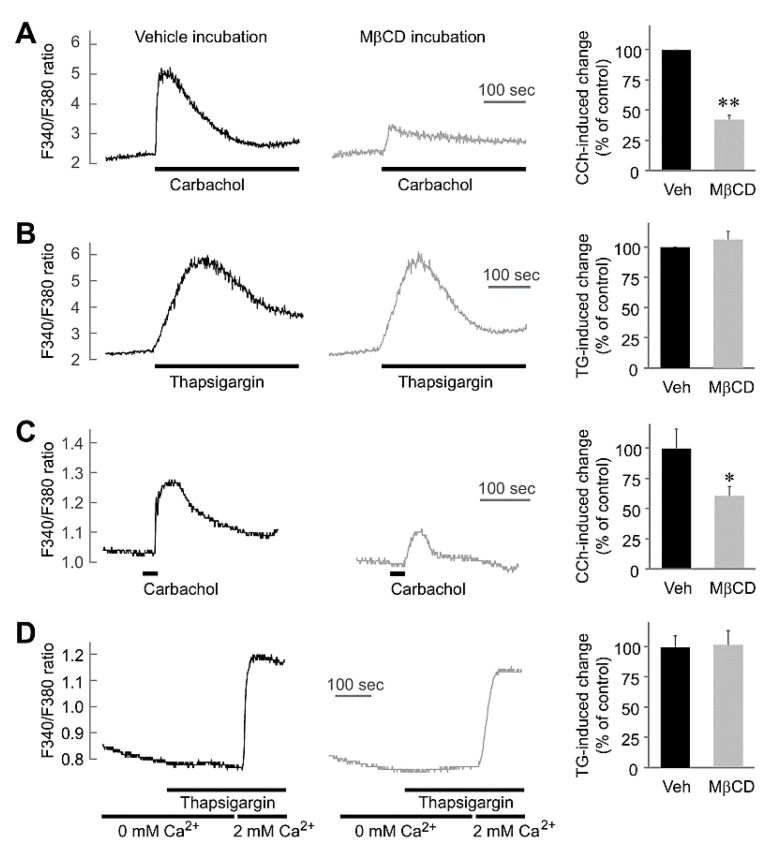
Preincubation with MβCD inhibits carbachol-induced Ca^2+^ increases but not thapsigargin-induced Ca^2+^ increases. (**A**,**B**) Fura-2/AM-loaded HSG cells were treated with 100 µM carbachol (**A**) or 1 µM thapsigargin (**B**) with (black trace, left) or without (gray trace, right) preincubation with 10 mM MβCD for 30 min. Typical Ca^2+^ transient experiments are shown. Quantification of F340/F380 changes (*n* = 3) induced by 100 µM carbachol or 1 µM thapsigargin preincubated with 10 mM MβCD. (**C**,**D**) Representative Ca^2+^ imaging traces in SMG cells isolated from mice. Cells were treated with 100 µM carbachol with (black trace, *n* = 27 cells) or without (gray trace, *n* = 27 cells) 10 mM MβCD preincubation for 30 min. Vehicle (black trace, *n* = 32 cells) or MβCD (gray trace, *n* = 30 cells) preincubation cells were incubated in Ca^2+^-free buffer and stimulated with 1 µM thapsigargin and then in 2.2 mM Ca^2+^-containing buffer. The results summary shows the percentage of all cells that responded to CCh or thapsigargin. Data are presented as mean ± SEM. * *p* < 0.05; ** *p* < 0.01.

We next investigated the effect of MβCD treatment on intracellular Ca^2+^ signaling in HSG cells. A 30-min preincubation with 10 mM MβCD inhibited the [Ca^2+^]_i_ increase triggered by carbachol (t_(2)_ = 16.37, *p* < 0.01; Figure 2A). However, MβCD incubation had no effect on the [Ca^2+^]_i_ increase induced by thapsigargin, a sarcoplasmic reticular Ca^2+^-ATPase (SERCA) inhibitor (t_(2)_ = 1.00, *p* = 0.42; Figure 2B). We confirmed these results in isolated mouse submandibular gland acinar cells. As in the HSG cells, the carbachol-mediated [Ca^2+^]_i_ increase was reduced in mouse submandibular acinar cells incubated with MβCD (t_(52)_ = 2.20, *p* < 0.05) (Figure 2C), whereas there was no difference in the thapsigargin-mediated [Ca^2+^]_i_ increase (t_(60)_ = 0.09, *p* = 0.93) (Figure 2D). Considering that thapsigargin depletes the intracellular calcium pool and induces SOCE, our results indicate that preincubation with MβCD suppresses only GPCR activation without affecting SOCE activity.

### 2.3. MβCD Preincubation Increased the BK Channel Activity in Salivary Gland Cells

**Figure 3:**
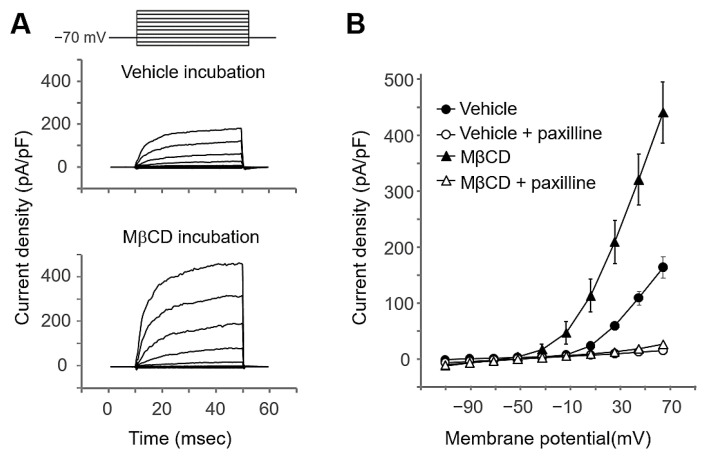
Preincubation with MβCD increases BK channel currents in mouse single SMG cells. (**A**) Typical current traces obtained in the control cells and those preincubated with 10 mM MβCD for 30 min. Currents were recorded immediately (15–20 s) after achieving whole-cell mode. (**B**) Average I–V relations of current amplitudes measured at the end of 40 ms pulses to the indicated potentials in the absence (●) or presence (▲) of 10 mM MβCD. Open circle (○) and open triangle (△) plots were obtained after the addition of 1 μM paxilline, a BK channel inhibitor. Each point represents the mean ± SEM (*n* = 10 or 11). The pulse protocol is shown as the graph insert.

When [Ca^2+^]_i_ is increased by GPCR activation in the salivary gland, a series of Ca^2+^-activated ion channels is activated. Large-conductance Ca^2+^-activated K^+^ (BK) channels are present in the cholesterol-enriched lipid raft domain [25]. We monitored changes in BK channel activity by cholesterol depletion in isolated mouse submandibular gland acinar cells using whole-cell patch-clamp recording. Interestingly, the preincubation of cells with 10 mM MβCD for 30 min increased K^+^ currents (Figure 3A), which were inhibited by the BK inhibitor paxilline (MβCD, F_(1,38)_ = 17.99, *p* < 0.001; paxilline, F_(1,38)_ = 59.41, *p* < 0.001; mV, F_(9,342)_ = 127.19, *p* < 0.001) (Figure 3B). These results imply that salivary BK channel activity is increased by lipid raft disruption.

### 2.4. MβCD Preincubation Did Not Directly Affect AQP5 Translocation in Salivary Gland Cells

**Figure 4:**
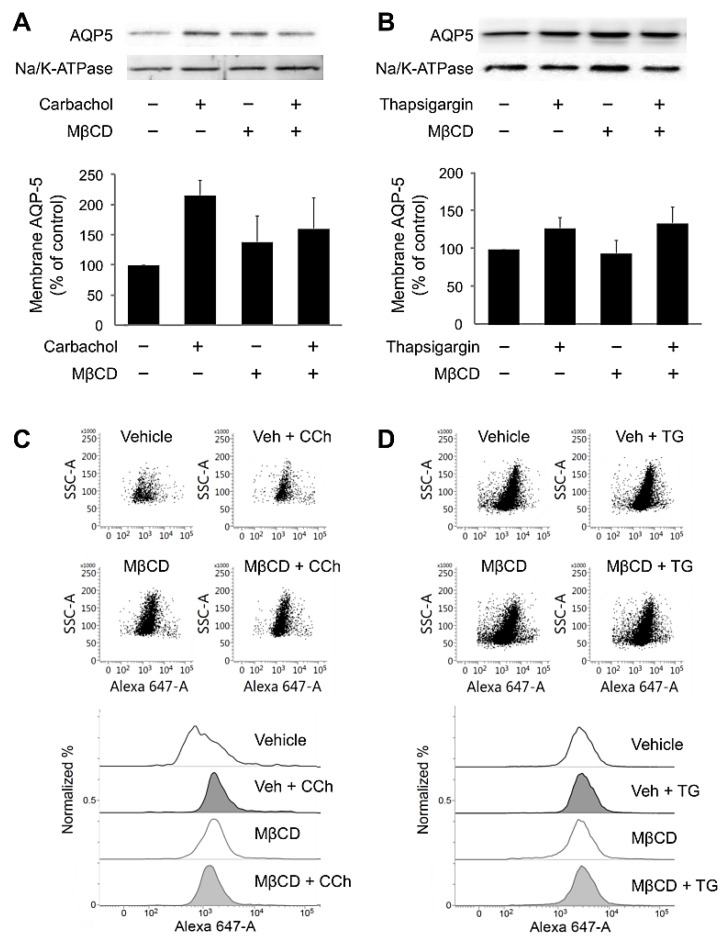
MβCD incubation does not directly affect AQP5 translocation. (**A**,**B**) HSG cells transfected with GFP-tagged AQP5 were preincubated in either the absence or presence of 10 mM MβCD for 30 min and then challenged with 100 μM carbachol (**A**) or 1 μM thapsigargin (**B**). (Top) Western blots show membrane AQP5 and Na^+^-K^+^-ATPase. (Bottom) Quantification of membrane AQP5 with Na^+^-K^+^-ATPase control (*n* = 3 to 6). n.s. = not significant. (**C**,**D**) Representative dot plots and histograms from flow cytometry analysis represent the surface expression of AQP5 in the mouse isolated SMG cell. The cells were treated with or without 10 mM MβCD preincubation for 30 min and then challenged with 100 μM carbachol (**C**) or 1 μM thapsigargin (**D**).

Muscarinic Ca^2+^ signaling triggers the membrane translocation of AQP5, a water channel protein in the salivary glands [22,26,27]. To determine the effect of MβCD on aquaporins, we analyzed the expression level of AQP5 via GFP intensity in the membrane fraction. In a previous study, we confirmed that 20 min of carbachol treatment was optimal for confirming activity-dependent AQP5 translocation [28]. We prepared the membrane fraction of HSG cells and analyzed the level of AQP5 in comparison with Na^+^/K^+^ exchanger, a membrane-enriched protein. We found that MβCD treatment marginally decreased the carbachol-induced AQP5 translocation (F_(3.8)_ = 1.82, *p* = 0.22; Figure 4A). Interestingly, MβCD treatment did not inhibit thapsigargin-induced AQP5 translocation (F_(3.8)_ = 1.60, *p* = 0.26; Figure 4B). A flow cytometry experiment showed that a similar manner of AQP5 translocation was observed in the mouse isolated SMG cells (Figure 4C,D). These results suggest that the MβCD-mediated change in the salivary AQP5 translocation was mainly due to the decrease in the muscarinic receptor-induced Ca^2+^ signaling.

## 3. Discussion

The physiological role of lipid rafts to optimize the functional cellular environment by maintaining membrane protein location and proximity between key functional players has received much scientific attention. In particular, the lipid raft mediates the physical association of GPCRs and downstream signal transduction factors. Thus, the lipid raft is more important in salivary cells that function based on polarity and those that function through several membrane events compared to their counterparts. However, the effects of the lipid raft on membrane events in salivary glands have not yet been clearly determined.

In this study, we aimed to understand the role of lipid rafts in salivary function. Muscarinic M3 receptor-mediated Ca^2+^ signaling plays an important role in AQP5 translocation in salivary gland cells [22,29]. The muscarinic mechanism in the plasma membrane includes M3 receptor activation, SOCE activation, and AQP5 translocation. In addition, muscarinic M1 and M3 receptors and (voltage-gated) K^+^ channels exist in the membrane lipid raft and functionally couple in neurons [11] and HEK cells [30]. In addition, a BK channel also exists in the lipid raft [31,32,33]. We think that the muscarinic receptors and BK channels of salivary gland cells also exist in the lipid raft. Our results show that MβCD-mediated lipid raft depletion inhibited carbachol-induced intracellular Ca^2+^ increase (Figure 2). The effect of lipid rafts on GPCR activation has been reported in many cells with frequent signal transduction (e.g., neurons). Lipid rafts have been reported to regulate N-methyl-D-aspartate receptor stabilization and receptor expression [34,35,36]. We also have reported that lipid rafts are important for AMPA receptor trafficking in a previous study [37]. Our results suggest that lipid rafts optimize GPCR signaling in salivary gland cells.

Interestingly, our findings reveal that the thapsigargin-triggered Ca^2+^ increase was not affected by MβCD (Figure 2C). Thapsigargin is a sarco/endoplasmic reticulum calcium-ATPase (SERCA) inhibitor that induces Ca^2+^ pool depletion by inhibiting recharge of the cytosolic Ca^2+^ pool and subsequently inducing SOCE without GPCR activation. Thus, our findings of different inhibitory effects on carbachol and thapsigargin imply that the lipid raft inhibits GPCR activation without affecting SOCE activity. However, there have been several previous reports that SOCE is affected by lipid rafts [38,39,40]. We suspect this discrepancy is due to the difference in SOCE characteristics. Despite the commonality of the triggering mechanism (i.e., intracellular Ca^2+^ pool depletion), SOCE characteristics depend on cell type. SOCE is mediated by a series of factors, including TRPC1, Orai1, and STIM1 [41]. We have previously observed that thapsigargin-induced SOCE in PC12 cells is different from those in HL-60 cells and Jurkat T cells [42]. Therefore, we concluded that lipid rafts do not affect SOCE in salivary gland cells.

We found that the disruption of lipid rafts, which caused a decrease in muscarinic receptor function in HSG cells (Figure 2), increased BK channel activity (Figure 3). It has previously been shown that cholesterol modulates BK channel activity by controlling lipid rafts [43]. The cholesterol depletion-mediated increase in BK channel current activity has been found in IGR39 human melanoma cells [32] and rat vascular smooth muscle cells [33,44]. It has been reported that cholesterol treatment reduces the open probability of the BK channel in rat cerebral artery myocytes [45]. However, it also has been reported that the BK channel current was decreased by MβCD in D54-MG and U251 glioma cell lines [31], implying that the relationship between lipid rafts and BK channel activity depends on cell type. Disruption of the cholesterol-enriched lipid raft structure is expected to modulate the activity by controlling the gating of the BK channel [25], but a clearer understanding of the molecular mechanism remains to be elucidated.

Finally, we examined the effect of lipid raft depletion on AQP5 translocation to the plasma membrane. Interestingly, MβCD inhibited carbachol-induced AQP5 translocation but did not affect thapsigargin-induced AQP5 translocation (Figure 3). Given that MβCD did not cause a thapsigargin-induced Ca^2+^ increase or an increase in AQP5 translocation, our results indicate that the lipid raft does not affect the translocation of AQP5. It has been reported that lipid rafts are involved in targeting aquaporins [46], and the colocalization of the lipid raft markers flotillin and AQP5 is increased during treatment with muscarinic receptor agonists and Ca^2+^ ionophores [19]. However, it is unclear how lipid rafts affect these sequential events in membranes (i.e., from muscarinic receptor activation to AQP5 translocation). Our results clearly show that MβCD inhibits carbachol-induced AQP5 translocation, mainly at the receptor level.

Lipid-lowering medications prescribed for hypercholesterolemia to lower blood cholesterol include HMG-CoA reductase inhibitors, commonly referred to as statins [47]. Interestingly, it has been reported that xerostomia, along with lichenoid and aphthae, is frequently found in patients receiving a statin prescription [48]. In addition, when statin treatment was stopped in dry mouth patients undergoing statin treatment, dry mouth symptoms were found to be relieved [49]. Our results, elucidating the relationship between cholesterol-depletion and salivary signaling, strongly suggest the causative mechanism of dry mouth in lipid-lowering medications.

Taken together, we examined the effects of lipid rafts on muscarinic signaling and found that lipid rafts regulate GPCRs and BK channels in the salivary gland cells. Our study will help others understand the molecular target of the lipid raft on the muscarinic signaling-mediated water secretion and will contribute to a better understanding of the cellular and molecular mechanisms of lipid rafts in modulating exocrine functions.

## 4. Materials and Methods

### 4.1. Reagents

Carbachol, MβCD, and thapsigargin were purchased from Sigma (St. Louis, MO, USA), and fura-2-acetoxymethyl ester (fura-2/AM) was obtained from Molecular Probes (Eugene, OR, USA). Dulbecco’s Modified Eagle Medium (DMEM), fetal bovine serum, and penicillin/streptomycin were purchased from GIBCO (Grand Island, NY, USA). The antibodies acquired from commercial sources were as follows: aquaporin-5 goat polyclonal IgG (Santa Cruz, sc-9891, 1:1000, Dallas, TX, USA), anti-alpha 1 sodium-potassium ATPase (Abcam, ab7671) primary antibody, donkey anti-goat IgG-HRP (Santa Cruz, sc-2020, 1:10,000), and goat anti-mouse IgG-HRP (Santa Cruz, sc-2031, 1:10,000) secondary antibody.

### 4.2. Cell Preparation

A human salivary gland (HSG) ductal cell line [50] was grown in DMEM supplemented with 10% (*v*/*v*) heat-inactivated fetal bovine serum and 1% (*v*/*v*) penicillin (5000 U/mL) and streptomycin (5000 µg/mL). The cells were cultured in a humidified atmosphere of 95% room air and 5% CO_2_. The culture medium was changed every two days, and the cells were sub-cultured weekly. Isolated mouse submandibular gland acinar cells were prepared as previously reported [17]. Briefly, mouse submandibular glands were surgically removed, finely minced with scissors, and digested for 5 min in 0.02% trypsin-EDTA (GIBCO) and 0.5 mg/mL collagenase (Sigma) in DMEM. The cells were triturated and centrifuged at 190× *g* for 1 min and then washed twice with serum-free DMEM. Acinar cells were attached to poly D-lysine-coated glass coverslips.

### 4.3. Cytosolic Free Ca^2+^ Measurement

The cytosolic free Ca^2+^ concentration ([Ca^2+^]_i_) was determined using the fluorescent Ca^2+^ indicator fura-2/AM, as previously described [27,28]. Briefly, the cell suspension was incubated in fresh medium containing fura-2/AM (4 µM) for 60 min at 37 °C with continuous stirring. Cells on coverslips were mounted onto the inverted microscope (Olympus IX70, Tokyo, Japan) and perfused continuously at 2 mL/min by a bath solution containing 140 mM NaCl, 5 mM KCl, 1 mM MgCl_2_, 2 mM CaCl_2_, 10 mM HEPES, and 10 mM glucose (pH 7.2). Fluorescence ratios were monitored with dual excitation at 340 nm and 380 nm and emission at 500 nm.

### 4.4. Cholesterol Measurement

Cellular and plasma membrane cholesterol levels were determined using the Amplex Red Cholesterol Assay Kit (Molecular Probes) based on an enzyme-coupled reaction that detects both free cholesterol and cholesteryl esters, according to the manufacturer’s instructions. In brief, a 50-μL sample was mixed with 50 μL Amplex Red Reagent containing 2 U/mL HRP, 2 U/mL cholesterol oxidase, and 0.2 U/mL cholesterol esterase and incubated at 37 °C for 30 min. Fluorescence was measured using an Epoch2 microplate spectrophotometer (Bio-Tek, Winooski, VT, USA) using an excitation and emission of 530 nm and 590 nm, respectively. 

### 4.5. Cell Viability Assay

Cell viability was determined using the trypan blue exclusion assay [24]. The HSG cells were treated with 10 mM MβCD for 30 min, harvested, and added to 0.4% trypan blue (Sigma). The percentage of viable (trypan blue-unstained) cells was measured by counting the cells under the microscope. All counts were performed in triplicate.

### 4.6. Electrophysiological Recording

Whole-cell patch-clamp recordings were obtained using a HEKA EPC-9 amplifier (HEKA Elektronik, Lambrecht, Germany). The patch pipettes (3–6 MΩ resistance) were filled with 135 mM K-glutamate, 5 mM EGTA, 3 mM CaCl_2_, and 10 mM HEPES, pH 7.2. The external solution contained 150 mM Na-glutamate, 5 mM K-glutamate, 2 mM CaCl_2_, 2 mM MgCl_2_, and 10 mM HEPES, pH 7.2. The stimulation protocol to generate current-voltage (I–V) relationships consisted of 40-ms voltage steps from −110 to +70 mV in 20-mV increments starting from a holding potential of −60 mV. Data were acquired and analyzed using PATCHMASTER software (HEKA Elektronik).

### 4.7. Preparation of Membrane Fractions and Western Blotting 

The HSG cells were transiently transfected with the pEGFP-C1-AQP-5 plasmid using Lipofectamine 2000 [27]. AQP-5-transfected HSG cells were grown in a 60-mm dish, pre-incubated with MβCD for 3 min, and treated with carbachol or thapsigargin for 20 min at 37 °C. The membrane fraction was obtained as previously described [28] and sonicated in ice-cold 20 mM HEPES solution containing 1 mM MgCl_2_, 100 mM NaCl, 1 mM dithiothreitol, and 0.3 mM phenylmethylsulfonyl fluoride (3 × 30 s; Sigma) at pH 7.4. The samples were centrifuged at 600× *g* at 4 °C, and the supernatants were centrifuged further at 20,000× *g* at 4 °C. The pellets (P2 membrane fractions) were separated via SDS-PAGE, and the proteins were transferred onto a PVDF membrane for immunoblotting. After blocking, the membrane was incubated with anti-AQP-5 (Santa Cruz, sc-9891, 1:200) as the primary antibody. The membrane was washed, incubated with donkey anti-goat IgG-HRP, and subjected to an electrochemiluminescence assay for detection. For normalization of the AQP5 signal, the membranes were stripped and re-probed with antibodies for anti-α1 Na^+^/K^+^-ATPase (Abcam, ab7671, 1:1000).

### 4.8. Analysis of AQP5 Protein Expression by Flow Cytometry

To evaluate the translocated AQP5 surface expression, we used flow cytometry in non-permeabilized cells performed as reported previously [51]. Briefly, mice SMG single cells were obtained by mashing using a 100 μm cell strainer, followed by washing the cell strainer twice with 5 mL of 0.5% bovine serum albumin (BSA) in PBS. The cells were then centrifuged at 190× *g* and washed twice with 0.5% BSA in phosphate-buffered saline (PBS). The cells were fixed with 4% formaldehyde, washed, and blocked 2% BSA in PBS. After blocking, the cells were washed and resuspended in 100 μL Alexa Fluor 647 conjugated-AQP5 antibody (Abcam, ab215225, 1:500), and incubated for 30 min at 4 °C in the dark. All of the washing steps were carried out using 1% FBS in PBS as washing buffer, followed by centrifugation (8000 rpm, 1 min, 4 °C). The labeled cells were kept on ice until analysis. The cell samples were then acquired on a FACSVerse flow cytometer equipped with FACSUITE software (BD Biosciences, San Jose, CA, USA).

### 4.9. Data Analysis

All quantitative data are expressed as mean ± SEM. Differences were determined by one-way analysis of variance (ANOVA) and considered significant when *p* < 0.05.
